# An evaluation of the accuracy of small-area demographic estimates of population at risk and its effect on prevalence statistics

**DOI:** 10.1186/1478-7954-11-24

**Published:** 2013-12-20

**Authors:** Jack D Baker, Adelamar Alcantara, Xiaomin Ruan, Srini Vasan, Crouse Nathan

**Affiliations:** 1 University of New Mexico, MSC06 3510, Albuquerque, NM 87131, Mexico

## Abstract

Demographic estimates of population at risk often underpin epidemiologic research and public health surveillance efforts. In spite of their central importance to epidemiology and public-health practice, little previous attention has been paid to evaluating the magnitude of errors associated with such estimates or the sensitivity of epidemiologic statistics to these effects. In spite of the well-known observation that accuracy in demographic estimates declines as the size of the population to be estimated decreases, demographers continue to face pressure to produce estimates for increasingly fine-grained population characteristics at ever-smaller geographic scales. Unfortunately, little guidance on the magnitude of errors that can be expected in such estimates is currently available in the literature and available for consideration in small-area epidemiology. This paper attempts to fill this current gap by producing a Vintage 2010 set of single-year-of-age estimates for census tracts, then evaluating their accuracy and precision in light of the results of the 2010 Census. These estimates are produced and evaluated for 499 census tracts in New Mexico for single-years of age from 0 to 21 and for each sex individually. The error distributions associated with these estimates are characterized statistically using non-parametric statistics including the median and 2.5th and 97.5th percentiles. The impact of these errors are considered through simulations in which observed and estimated 2010 population counts are used as alternative denominators and simulated event counts are used to compute a realistic range fo prevalence values. The implications of the results of this study for small-area epidemiologic research in cancer and environmental health are considered.

## Introduction

In recent years, a growing demand for small-area demographic estimates has been observed. Much of this demand comes from epidemiologists, who utilize these estimates for small-area surveillance efforts in the areas of cancer and environmental epidemiology in particular [1-5]. The potential of small-area epidemiology has generated considerable excitement [1-5]; however, it has also created important challenges for the demographers who produce small-area estimates of population at risk as well as the epidemiologists who use them. At a fundamental level, it is well known that as the size of the population to be estimated decreases, errors in demographic estimates increase [6-11]. These errors can be surprisingly large [6-11], but at present their impact on small-area epidemiologic measures has been incompletely described, and the implication of these errors for small- area health tracking and analytic epidemiology has not received an adequate amount of attention [12-17]. This paper attempts to fill this gap by characterizing the errors associated with a set of single-year- of-age estimates made at the level of United States census tracts and analyzing the potential sensitivity of small-area crude prevalence measures to these errors.

This example is extreme in both its spatial scale (census tracts represent very small areas, often a single neighborhood) [18] as well as in the fine-grained age intervals to be estimated. Errors in census tract-level estimates in five-year age groupings reported in previous studies have ranged between as small as 10% [19] and as high as 80% or more [9]. It is known that single-year-of-age estimates can be relatively more volatile than those constructed in five-year age intervals [11,20]. A number of methods exist for making single-year-of-age estimates. Assuming monotonicity within five-year age intervals [21,22] and the stability of demographic processes over these short time intervals [18], demographers have historically made use of methods that break out five-year interval estimates into single years of age through pro-rating, osculatory interpolation, or the closely related procedure known as “spline-fitting” [11,20-30]. Pro-rating involves the allocation of the five-year data based on either historical or assumed proportions; for example, one might divide five-year estimates into single years based on the known distribution of the last census or based on an assumption of rectangularity (equal proportions of one-fifth) [11]. Osculatory interpolation, in contrast, relies upon a theory in mathematics that revolves around the unique solution of simultaneous equations using linear systems designed to minimize discrepancies between observed five-year data and the re-aggregation of single- year-of-age estimates into corresponding intervals [11,20,22-30]. Spline-fitting, similar to osculatory interpolation, involves the overlapping of multiple polynomials to arrive at estimates of distributions through an optimization component based on the least-squares criteria [31]. The first two procedures have been the most widely applied within applied demography; a rather long historical discussion of spline-fitting has not resulted in its general implementation by demographers working in non-academic settings (such as state government) where functionally utilized population estimates are typically made.

The purpose of this paper is not to contrast the accuracy of these methods; rather, we seek to implement commonly utilized methods to characterize the magnitude of errors associated with a typical set of estimates of population at risk likely to be utilized by small-area epidemiologists in practice. The focus, therefore, will be upon describing the range of errors that one might expect to see in such a set and analyzing how these errors might impact a set of crude-prevalence estimates made at a correspondingly fine-grained spatial scale (census tracts). To accomplish this purpose, data from the 2010 US Census are extracted (Summary file 1) for all census tracts (n = 499) within the state of New Mexico extracted from the American Factfinder website—[32]. The data extracted include a gold-standard set of single-year-of-age counts and the corresponding five-year grouped data for each census tract. Our evaluation is straightforward: we compare single-year-of-age estimates made using methods of pro-rating and osculatory interpolation of five-year grouped data to observed single-year-of-age 2010 Census counts and characterize the moments of the resulting ex-post facto error distributions using established methods within demography [6,8,10]. Next, we simulate a range of plausible event prevalences using published estimates of childhood obesity rates and use them to analyze the effects of observed errors in demographic estimates on estimates of prevalence per 1,000 person-years. The results are considered in light of practice in small-area epidemiologic surveillance and suggestions for further research and evaluation are made.

## Materials and methods

### Input data and study area

New Mexico represents a diverse study area where tract-level variation in population characteristics can vary dramatically in concordance with larger geographic trends at the county level. The state is characterized by highly urbanized and rapidly growing metropolitan areas such as the cities of Las Cruces, Rio Rancho, and Albuquerque, dynamic and steady-growing small towns such as Roswell, Alamogordo, Clovis, and Farmington (just to mention four), vast sections of rural areas and the presence of 22 tribal groups with long-standing historical presence in the state, numerous Colonias [3], and by an overlapping mosaic of historical Land Grant Communities linked to the Spanish Colonial Era and the period of Mexican Independence prior to New Mexico becoming a US territory in 1850 at the conclusion of the Mexican-American War. To review, New Mexico represents a microcosm of the demography of many communities throughout the United States as well as important and distinctive populations. Each of these dynamics will be represented at the Census tract level, providing substantial heterogeneity and material for analysis in the current context. Counts of age/sex-specific population in five-year intervals (0 to 4, 5 to 9, 10 to 14, 15 to 19, 20 to 24) and in single years (0 to 21) were extracted from the SF1 file from the 2010 Census. Data were extracted at the census tract level (n = 499) for the entire state of New Mexico. Data were not considered for specific race/ethnicity group, with the data focused only on “all race” counts.

### Pro-rating and interpolation in demography

In demography, the term “pro-rating” refers to the allocation of grouped data into more fine- grained categories, such as decomposing five-year age-grouped data into single years as in the current analysis [11,20]. In this study, pro-rating serves as a baseline activity--simpler than the methods of polynomial interpolation described below but also dependent upon specific assumptions with little appealing mathematical theory underlying them [23,24]. Here, rectangular pro-rating is utilized in which the assumption is made that single-year age groups within any five-year age interval are equivalent: each single-year comprises one-fifth of the five-year age-grouped data [11]. As pointed out by Brass [23] and others [11,20] this method assumes that population processes—such as birth, death, and migration functions—are similar from year to year within the five-year age interval in question [21,22], i.e., that the single year data are monotonic in relation to the five-year grouped data they produce [21,22]. This simplifying assumption is unlikely to be true, and rectangular pro-rating is generally considered as a strategy to be implemented when no ancillary information on population dynamics is available at an appropriate geographic level [11,20].

**Figure 1:**
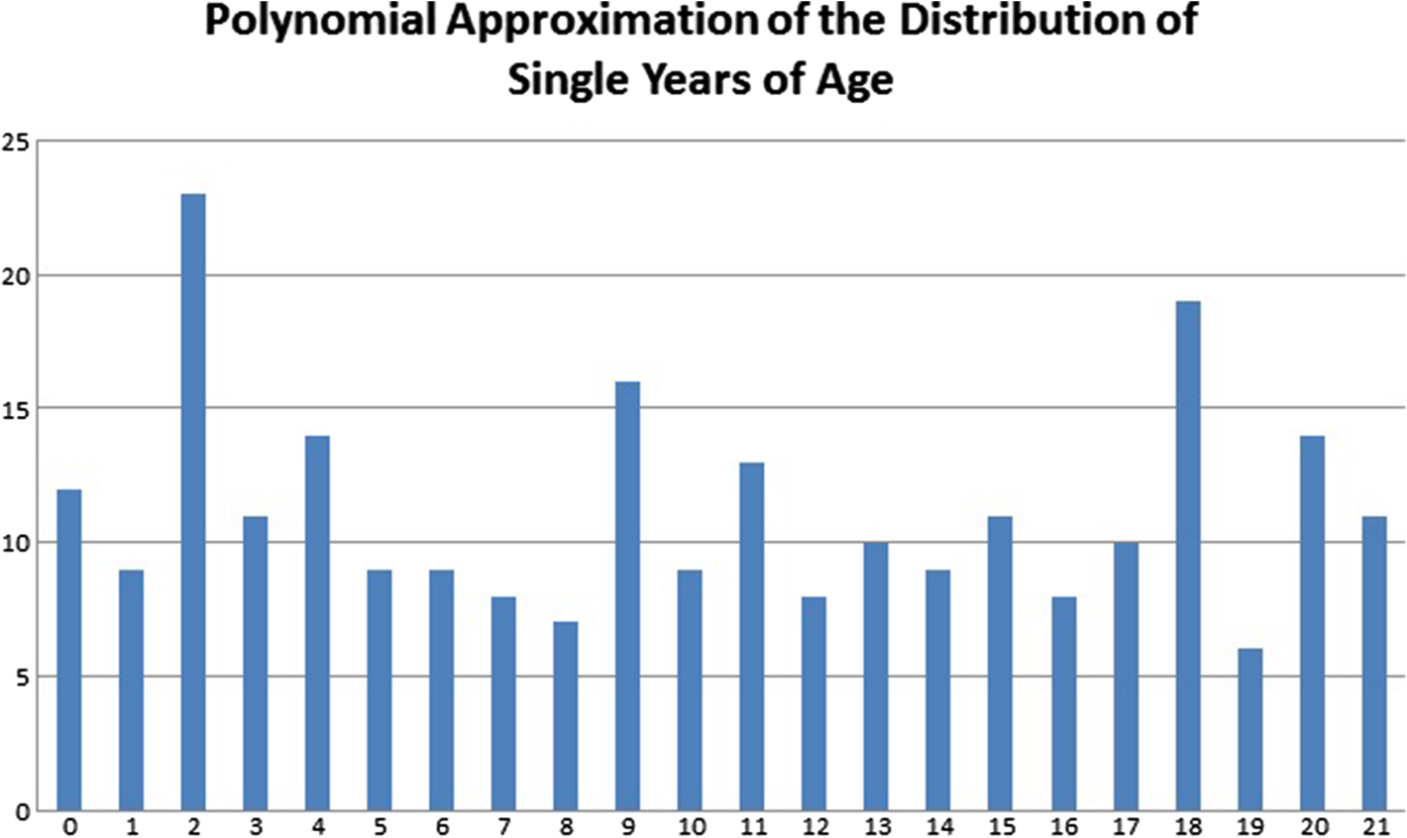
Polynomial approximation of the distribution of single years of age.

The use of polynomial functions to describe relationships between time-ordered inputs and function-generated outputs has a long history within mathematics [33,34]. Their use in generating intermediate and unknown values within a dataset by interpolating between known values has an equally long history in applied fields such as climatology, economics, and demography [11,20-30]. Though polynomial interpolation approaches have been criticized in demography as being blind to population theory [20,23,24], in practice interpolation is easy to implement as many standardized formulas have been presented that involve only “plugging-in” of demographic data grouped in five-year intervals into predefined formulae to arrive at single-year-of-age estimates [11]. Figure 1 illustrates the relationship between single-year age structure and a polynomial function used to decompose five-year grouped data.

As in Figure 1, an nth degree polynomial of the form:

$$y=A+\mathrm{Bx}+{\mathrm{Cx}}^{2}+{\mathrm{Dx}}^{3}+\ldots +{\mathrm{Nx}}^{n}$$

may be fit to any curve for which some data points are known with certainty to arrive at estimates of intermediate values. We may think of the interpolating polynomial as a system of equations, represented in terms of the well-known Vandermonde matrix (representing known values of demographic data), premultiplied against a vector of coefficients A to An, to yield interpolated values Yi as in the linear equation. Once solved, the function defined to estimate the yi is known as the interpolant [18,19,21]. It is known that higher-degree interpolating polynomials may often provide poorer fit of intermediate points, suggesting that simpler polynomial interpolants utilized by demographers may, in fact, provide more accurate estimates of single years of age [11,34,35].

Exact solutions to such approximating polynomials are difficult to implement using demographic data in five-year age groups [18,19,21]; however, their approximation through differencing formulas--those that minimize differences between five-year grouped counts and estimated values thereof using a polynomial function are well known and highly accurate in implementation [3]. An example is the Lagrange formula (from reference [11], page 683):

$$\begin{array}{l} f\left(\times \right)=f\left(a\right)\;\left[\left(\left(\times -b\right)\left(\times -c\right)\left(\times -d\right)/\left(a-b\right)\left(a-c\right)\left(a-d\right)\right)\right]+\\ f\left(b\right)\;\left[\left(\left(\times -b\right)\left(\times -c\right)\left(\times -d\right)/\left(a-b\right)\left(a-c\right)\left(a-d\right)\right)\right] \end{array}$$

which fits a polynomial of the form presented and passing through the two points a and b (which in this case are five-year grouped age counts) by minimizing differences between estimated values from the polynomial functions and these observed counts by shifting the values of the constants A, B, C, D, etc. [11,34]. In practice, the fitting of points f (x) are accomplished by inputting values of f (a) and f (b) into established formulas. This example of a Lagrangian polynomial passing through two points may be generalized to as many points as desired, and various methods of interpolation in demography rely upon differing numbers of points to achieve the desired fit. Osculatory interpolation is similar to the method of spline-fitting, also utilized in demography [21,22]; here we choose to focus on several methods of osculatory interpolation as better representing methods that are more typically used in practice among applied demographers. This choice does not reflect methodological preference, but better suits the purpose of this paper, which is to characterize the magnitude of errors that practicing epidemiologists and demographers might expect to see in small-area, fine-grained (with respect to age) estimates of population at risk and their impacts on measures of epidemiologic risk.

In this paper, we utilize several commonly implemented osculatory interpolation procedures including: the Karup-King [25,30], Beers 1 [11], Beers 2 [26], and Sprague methods [29]. These methods differ in the number of points taken in the interpolation, with the Karup-King taking two differences, the Beers 1 and 2 focusing on four and six differences, and the Sprague method relying upon five. In general, previous studies in other fields [34,35] have suggested that the use of fewer points might enhance local accuracy in the interpolation [11,23-30], leading to a general hypothesis that the Karup-King may tend to out-perform alternatives.

### Statistical comparisons of error and model evaluation criteria

Percentage discrepancies between the single-year-of-age estimates and corresponding 2010 Census counts form the basis of the evaluation reported in this paper, in accordance with the ex-post- facto evaluation method typically utilized by demographers [6-11]. Because demographic error distributions are calculated across geographic levels with widely differing population sizes, the use of percentage error is often encouraged [8,36] and is therefore employed here. Demographic estimate error distributions are characterized by non-normality and a frequent lack of symmetry [8,35], making it difficult to make statements about the range of variation in estimation accuracy or to determine what is or is not an extreme error value [8,10]. In this study, all statistical error distributions were found to deviate from normality using the Kolmogorov-Smirnov test at the alpha = 0.05 level. A simple non- parametric solution is to make use of the median as a summary measure of error and to utilize the percentile distribution between the 2.5th percentile and 97.5th percentile [37,38] to characterize precision; this is the strategy employed in this paper. These summary measures are computed for each age/sex group, as well as across the entire range of ages within each sex. While this approach makes sense in light of the nature of the statistical error distributions employed in demography, there is a lack of consensus in the literature about what constitutes a “better” estimate among available alternatives [8,10]. The perspective taken in this paper is to evaluate how much better one might do by employing a polynomial interpolation method than they would do by using a naive model based on simple rectangular pro-rating (assuming that one-fifth of the five-year age/sex count is within each single-year-of-age interval). This is the approach taken by Harper, Coleman, and Devine [39] as well as by Swanson and Tayman [40] in their “proportionate reduction in error” statistic. Because this paper relies upon summary statistics based on percentages, models are evaluated in terms of: (1) the improvement in percentage point error observed in each age/sex interval and (2) by the percentage point range between the 2.5th and 97.5th percentiles of the error distributions. The “best” fitting model, then, is determined to be the model that results in the greatest improvement in percentage accuracy over rectangular pro-rating and the lowest range of values between the 2.5th and 97.5th percentiles of the error distribution.

Previous studies [9,19] of errors associated with demographic methods at the census tract level have indicated that over 10-year periods starting at the previous census, a substantial amount of error may accumulate [8,41]. Errors in these studies have ranged between as low as 10% and as high as 80% within any age/sex five-year age grouping. For single-year-of-age estimates, it could be anticipated that errors could be larger than this, but isolating how much of this error would be due to the practices of pro-rating or polynomial interpolation would be difficult since errors in the five-year age/sex-grouped estimates would also affect the single-year-of-age estimates. To avoid this challenge, in this study we utilize polynomial interpolation and pro-rating methods on known 2010 Census five-year counts. This practice isolates the error associated with the method by eliminating the conflation associated with using uncertain five-year age/sex-specific estimates. The errors and error distributions reported in this study are due solely to those associated with the methods of pro-rating and polynomial interpolation that are the focus of the paper.

### The effect of errors in small-area demographic estimates on epidemiologic statistics

Small-area epidemiology faces significant challenges in the geographic positioning of event data, through the process of geocoding [42-47], necessary for calculating epidemiologic statistics such as incidence, prevalence, etc. These issues should also be anticipated to be important in making inferences associated with analytic epidemiology [1-5,12-18,48-50], but they are beyond the scope of the current paper, which will examine only the effects of small-area demographic estimation error on surveillance statistics. To assess the impacts of errors in demographic estimates, the paper used a simple simulation-based approach to analyze the sensitivity of small-area crude prevalence estimates within each single-year-of-age grouping. The “best-performing” set of demographic estimates for each sex is utilized as a denominator in calculating risk measures. Event counts were simulated using childhood obesity (a common event whose prevalence has been estimated to be as high as 1/5 or 200/1000 persons) as an example. The distribution of prevalences was estimated using a Monte-Carlo simulation [51,52] in the R statistical package that assumed: (1) normality and symmetry of the prevalence distribution, (2) an average prevalence of 17.5%, and (3) a standard deviation of 2.5%. This distribution was resampled 10,000 times, with a burn-in period of 500 iterations and thinning to include only every 100th observation to avoid commonly known challenges related to autocorrelation of randomly generated number algorithms [51,52]. The resulting distribution of prevalence was used to estimate the 2.5th and 97.5th percentiles for use in the simulation. These points were then used to simulate case events for each census tract/sex/age grouping. Median differences between crude prevalence estimates of risk per 1,000 person-years calculated using 2010 Census counts and the demographic estimates of population at risk as alternatives were computed. Variability in terms of the errors associated with risk per 1,000 person-years were then assessed using the 2.5th and 97.5th percentiles in light of observed non-normality and asymmetry in the distributions of these differences.

## Results

### Errors in single-year-of-age small area estimates of population at risk

For males, the simplest interpolation method--the Karup-King procedure--produced the smallest errors for the most age groups. For nine out of 21 age intervals, this method was found to be the most accurate available method (Table 1). Use of this method would reduce error in comparison to the rectangular pro-rating method by as much as 46.30 percentage points (age 16) or as little as only 0.18 percentage points (age 6). On average, use of the Karup-King method would improve estimation accuracy by 7.82 percentage points over the rectangular pro-rating method. It is worth noting that the performance of the Karup-King method is similar to that of either the Beers 1 or Beers 2 methods, meaning that the sensitivity of epidemiologic statistics to a typical set of demographic estimates may be similar even when different methods are utilized—especially when we consider that each of these methods differs widely in the number of data points used in the interpolation. In contrast, the Sprague method provided much less reduction in error on average when compared to rectangular pro-rating (4.49 percentage points) and in a number of cases (ages 0, 4, 6, 9, 11, 14, and 21) actually provided a less accurate estimate than that observed when using simple rectangular pro-rating. Some of the increases in error are substantial when using the Sprague method, suggesting that the increased degree of this polynomial may be associated with poorer fitting of intermediate values as noted in studies in other fields [34,35]. These results appear to hold for males in terms of the precision of the methods. While all methods showed a wider range of error percentile distributions than is desirable (frequently difference between the 2.5th and 97.5th percentiles exceeded100 percent), the Karup-King likewise was consistently the smallest (median = 131.59 percentage points, lowest = 77.74, highest = 164.44). In 18 out of 21 cases, the Karup-King estimates were more accurate than using simple rectangular pro-rating.

**Table 1:** Summary characteristcis of percentage errors in estimates of single-years of age based on pro-rating or interpolation (males)

**Age**	**Rectangular**			**Karup-King**			**Beers1**			**Beers2**			**Sprague**		
	**Median**	**2.5 percentile**	**97.5 percentile**	**Median**	**2.5 percentile**	**97.5 percentile**	**Median**	**2.5 percentile**	**97.5 percentile**	**Median**	**2.5 percentile**	**97.5 percentile**	**Median**	**2.5 percentile**	**97.5 percentile**
0	9.28	-31.38	77.71	4.53	-32.72	82.29	5.86	-40.23	98.03	3.66	-43.73	79.96	20.77	-27.93	124.67
1	16.28	-33.83	111.44	7.02	-27.46	115.63	6.96	-35.88	117.87	7.17	-40.02	117.20	11.12	-33.48	127.63
2	7.00	-29.53	95.54	1.58	-25.32	93.97	0.91	-31.11	89.80	1.91	-28.97	93.95	-2.06	-33.00	84.93
3	12.28	-34.06	93.80	5.03	-27.9	104.69	4.22	-33.06	108.01	5.44	-32.28	104.07	-3.20	-43.05	86.88
4	3.45	-29.75	67.73	-1.42	-30.28	66.95	-3.30	-35.90	82.57	-1.50	-34.78	71.10	-10.72	-45.24	66.42
5	12.19	-40.89	136.25	3.87	-32.14	116.27	3.01	-41.60	118.33	4.02	-39.06	118.00	-4.80	-46.09	109.71
6	3.46	-40.96	81.71	-3.29	-34.00	81.02	-2.94	-39.86	81.19	-2.92	-39.03	77.86	-7.20	-42.97	74.27
7	9.89	-37.06	110.20	0.65	-33.26	114.93	1.57	-38.71	114.34	1.94	-39.92	113.03	1.20	-38.37	111.66
8	7.53	-39.27	90.23	-0.83	-38.28	98.04	-0.43	-43.11	100.97	-1.52	-41.61	90.33	3.49	-39.89	104.65
9	8.64	-37.54	103.83	1.77	-33.23	109.85	1.27	-42.80	109.62	1.67	-42.38	106.80	-73.16	-120.31	7.68
10	8.43	-43.84	102.00	0.19	-29.76	84.63	-0.52	-39.90	86.21	-0.82	-37.24	86.36	10.97	-31.20	111.89
11	11.17	-44.08	110.96	4.52	-35.41	114.06	3.48	-46.28	106.07	4.58	-42.90	111.96	23.82	-33.19	148.32
12	9.48	-38.93	105.83	0.13	-31.86	103.10	1.76	-43.29	105.89	1.84	-37.65	109.64	15.68	-31.99	137.70
13	9.82	-42.08	106.20	4.27	-36.57	114.20	5.35	-44.17	110.10	5.06	-41.11	111.60	-1.72	-48.92	98.58
14	9.93	-40.39	94.73	2.55	-43.37	121.07	6.62	-44.42	111.45	6.36	-42.92	111.21	-33.65	-64.20	26.61
15	23.60	-39.50	155.65	8.46	-46.60	114.63	9.94	-38.53	119.25	9.53	-37.66	120.46	26.75	-40.84	168.68
16	47.30	-40.61	162.86	-1.00	-26.93	65.02	8.42	-35.54	158.79	3.68	-36.85	136.91	3.78	-28.41	83.00
17	30.17	-34.66	164.14	3.26	-31.41	87.35	7.58	-35.68	164.13	8.71	-33.85	162.80	-1.56	-43.28	82.18
18	45.32	-36.48	114.29	-1.15	-29.55	54.49	5.43	-40.79	135.05	3.99	-38.22	138.66	-10.71	-45.76	47.68
19	34.83	-35.66	177.50	1.88	-30.52	100.87	6.84	-44.56	248.20	6.96	-43.93	239.47	-8.38	-60.73	87.21
20	21.96	-62.84	317.68	-3.40	-25.60	52.14	2.17	-48.13	238.03	1.74	-48.45	243.14	-10.26	-46.19	36.94
21	0.26	-60.00	340.75	-0.25	-36.62	78.02	-1.54	-52.60	280.56	-1.32	-53.54	286.11	-5.02	-48.93	73.72

Among females (Table 2), much less clear differences were observed in estimation accuracy across the available methods. All of the interpolation-based methods out-performed rectangular pro-rating in most cases: 16/22 with Karup-King, Beers 1 and Beers 2, and 13/22 for the Sprague method. The average reduction in error across the ages was greatest for the Beers 2 procedure, which reduced errors by over 4% on average; however, the reductions in error were within 1 to 1.5 percentage points across all of the alternatives. It is noteworthy, however that the specific ages in which each method performed best and the magnitude of reductions at each age across the methods varied. The only estimates that appeared to significantly increase bias were those made with the Sprague interpolants (as was observed in males), which increased errors by 65 percentage points among 9-year-old females and by 39 points among 14-year-olds. Overall, the range of errors associated with each procedure were extremely similar, though the Beers 2 procedure again out-performed very marginally. For the Beers 2 procedure, the difference between the 2.5th and 97.5th percentiles ranged from a low of 99.84 percentage points (13-year-olds) to a high of 239.22 percentage points (16-year-olds).

**Table 2:** Summary characteristics of percentage errors in estimates of single-years of age based on pro-rating or interpolation (females)

**Age**	**Rectangular**			**Karup-King**			**Beers1**			**Beers2**			**Sprague**		
	**Median**	**2.5 percentile**	**97.5 percentile**	**Median**	**2.5 percentile**	**97.5 percentile**	**Median**	**2.5 percentile**	**97.5 percentile**	**Median**	**2.5 percentile**	**97.5 percentile**	**Median**	**2.5 percentile**	**97.5 percentile**
0	5.24	-55.00	120.00	-4.80	-54.89	143.03	-2.98	-64.60	140.04	-5.54	-68.17	140.19	10.64	-52.52	171.40
1	6.43	-51.63	107.14	-1.39	-50.11	115.24	-1.50	-54.54	117.31	-1.64	-54.12	117.51	2.39	-50.51	121.98
2	5.74	-53.90	110.43	-2.28	-47.15	109.13	-3.27	-54.22	109.51	-2.13	-53.87	110.32	-6.12	-57.83	107.11
3	11.99	-46.80	142.78	3.52	-40.53	133.83	2.54	-49.59	126.65	3.80	-48.24	147.04	-4.86	-57.06	126.31
4	11.21	-52.30	156.83	-0.03	-40.49	135.02	-0.37	-48.10	13534	1.10	-45.65	137.51	-7.37	-56.81	124.70
5	8.07	-41.94	91.98	1.93	-39.26	98.74	1.94	-50.73	104.67	2.38	-47.82	104.86	-5.23	-58.29	93.99
6	4.77	-44.66	98.36	-3.02	-41.16	106.23	-3.16	-47.49	107.20	-3.00	-45.52	103.03	-7.04	-51.00	99.87
7	9.79	-43.72	111.58	0.58	-40.20	112.41	0.45	-45.26	125.21	0.95	-43.39	105.69	0.49	-44.60	111.41
8	-0.72	-47.39	78.57	-5.21	-40.72	70.79	-5.18	-44.64	71.45	-6.67	-46.44	67.19	-1.86	-41.48	74.83
9	7.05	-45.33	113.07	-0.15	-35.08	106.77	-0.62	-42.11	102.69	-0.47	-43.56	105.33	-72.83	-133.58	-32.05
10	-0.90	-41.00	76.98	-6.05	-39.12	94.82	-7.26	-44.71	83.96	-6.39	-43.77	84.55	4.11	-35.42	109.72
11	4.43	-37.85	92.87	-3.94	-34.85	90.57	-5.34	-42.50	89.47	-4.34	-37.71	91.71	12.24	-26.93	128.00
12	-4.83	-53.24	71.06	-10.60	-44.51	68.97	-10.50	-55.49	75.22	-9.98	-49.91	67.56	1.60	-47.79	96.63
13	-1.95	-40.00	70.45	-6.70	-38.40	68.52	-5.50	40.32	66.96	-4.43	-36.26	63.58	-12.50	-45.61	56.92
14	0.27	-56.50	87.48	-5.21	-52.31	90.57	-5.12	-52.52	85.92	-4.75	-51.65	85.33	-39.66	-73.16	20.13
15	5.56	-32.03	80.00	-2.82	-54.05	85.89	-0.48	-39.19	88.39	-0.46	-39.16	88.66	15.01	-48.14	113.38
16	14.99	-58.23	157.67	4.00	-58.21	139.07	10.42	-62.22	178.90	6.09	-67.18	172.05	8.49	-71.40	143.48
17	19.23	-55.82	148.02	8.91	-56.51	150.10	9.35	-58.16	151.78	9.40	-55.82	153.00	4.78	-74.34	143.84
18	19.70	-62.80	179.03	2.92	-68.88	186.40	5.93	-60.54	162.52	3.87	-59.42	168.77	-4.80	-86.64	180.27
19	21.00	-57.88	163.57	8.91	-58.52	149..85	8.49	-46.05	132.31	7.75	-46.20	129.38	-2.63	-85.99	145.00
20	9.05	-50.10	108.67	10.31	-65.92	237.83	8.07	-52.24	172.49	8.02	-52.36	161.94	1.42	-88.64	244.10
21	9.39	-29.52	106.67	12.76	-63.30	277.40	8.25	-30.33	124.11	7.48	-28.39	119.77	6.83	-80.50	266.51

A striking feature of the results is that demographic estimates of single-year-of-age population at risk at the Census tract level appear to be similar across the different methods utilized and to contain a surprising level of inaccuracy and a very large range of values across the set. We defined the “best” set as the alternative with the greatest reduction in error over simple rectangular pro-rating and the least observed spread between the 2.5th and 97.5th percentiles of the error distribution. The best-fitting set of estimates were utilized in analyzing the sensitivity of small-area crude prevalence measures to errors in these estimates. The best-fitting set for males was the Karup-King (two differences), while the Beers 2 (six differences) was utilized for females.

### Impact of errors on crude prevalence estimates

The effect of demographic estimation errors (Table 3) are relatively small at the lower end of the prevalence spectrum (2.5th percentile), on average never accounting for more than a difference of a few people in a crude prevalence estimate indexed at 1,000 person-years. Though the differences vary between the sexes in terms of the specific ages in which the larger errors are observed, similar differences in general were observed for male and female estimates. For males, median differences ranged from a high of -10 persons per 1,000 person-years to a low of effectively 0. Similarly, among females the highest observed median error was 14 persons and the lowest also effectively 0. The observed error distributions in both sets were asymmetrical, with a very large amount of variability observed in terms of the range of effects of observed. This is due both to high variability and the presence of notable outliers in both sets. Among male single-year-of-age estimates, the difference between the 2.5th and 97.5th percentiles ranged from a low of 99 persons per 1,000 person-years to a high of 210 persons per 1,000 person-years. Among females, even greater large-scale variability was observed with differences between the 2.5th and 97.5th percentiles ranging from a low of 145 to a high of 334.

**Table 3:** Percent-point impact of estimation errors, 2.5th percentile of stimulated prevalence

**Male**	**Age**	**Difference/1,000 Median**	**Difference/1,000 2.5th percentile**	**Difference/1,000 97.5th percentile**	**Female**	**Age**	**Difference/1,000 median**	**Difference/1,000 2.5th percentile**	**Difference/1,000 97.5th percentile**
	0	-6	-58	92		0	0	-56	89
	1	-8	-67	75		1	6	-76	219
	2	-2	-60	53		2	2	-67	147
	3	-6	-64	61		3	3	-65	146
	4	2	-50	61		4	-5	-74	116
	5	-5	-67	73		5	-1	-72	105
	6	4	-56	82		6	-3	-64	114
	7	-1	-67	85		7	4	-63	104
	8	1	-62	98		8	-1	-66	91
	9	-2	-65	86		9	9	-50	108
	10	0	-57	68		10	0	-64	91
	11	-5	-66	91		11	8	-60	96
	12	0	-63	74		12	6	-60	75
	13	-5	-66	108		13	14	-50	124
	14	-3	-68	142		14	6	-48	71
	15	-10	-67	135		15	6	-57	134
	16	1	-49	55		16	1	-59	80
	17	-5	-59	71		17	-7	-79	255
	18	1	-48	64		18	-11	-75	158
	19	-2	-63	69		19	-5	-78	183
	20	4	-44	54		20	-9	-40	107
	21	0	-56	89		21	-9	-77	137

At higher levels of simulated prevalence (97.5th percentile), both the median differences and the range of values between the 2.5th and 97.5th percentiles were both observably larger (Table 4). While this may be accounted for by the differences in the frequency of events (the 2.5th percentile of the simulated prevalence distribution is 12.57% and the 97.5th is 22.47%--amounting to nearly a 10 person difference per 1,000 person-years), the observations are striking. Errors range among males from a low of effectively zero to a high of -18 persons per 1,000 person years. Similarly, among females errors range from between a low of effectively 0 to a high of 25 persons per 1,000 person-years. In both cases, the range of differences per 1,000 person-years is nearly double that observed among the lower prevalence-based estimates. Among males, the errors range between a low of 178 persons per 1,000 person years to a high of 378 persons per 1,000 person years. Among females the differences between the 2.5th and 97.5th percentiles are even larger, ranging between a low of 215 persons per 1,000 person years to a high of 601 persons per 1,000 person-years.

**Table 4:** Percent-point impact of estimation errors, 97.5th percentile of stimulated prevalence

**Male**	**Age**	**Difference/1,000 median**	**Difference/1,000 2.5th percentile**	**Difference/1,000 97.5th percentile**	**Female**	**Age**	**Difference/1,000 median**	**Difference/1,000 2.5th percentile**	**Difference/1,000 97.5th percentile**
	0	-11	-104	165		0	0	-101	161
	1	-15	-118	135		1	12	-137	395
	2	-3	-109	96		2	4	-121	265
	3	-11	-115	110		3	5	-118	262
	4	3	-90	110		4	-8	-134	209
	5	-8	-121	131		5	-2	-130	189
	6	7	-101	148		6	-5	-115	206
	7	-1	-120	153		7	7	-114	188
	8	2	-111	176		8	-2	-118	163
	9	-4	-118	155		9	16	-90	195
	10	0	-103	122		10	1	-116	164
	11	-10	-120	163		11	15	-107	173
	12	0	-114	134		12	10	-107	136
	13	-9	-120	194		13	25	-91	224
	14	-6	-123	255		14	10	-87	128
	15	-18	-120	243		15	11	-103	240
	16	2	-88	99		16	1	-106	144
	17	-9	-107	129		17	-13	-142	459
	18	3	-86	116		18	-19	-136	284
	19	-4	-113	125		19	-8	-141	329
	20	8	-80	98		20	-16	-127	193
	21	0	-101	161		21	-17	-139	247

## Discussion

To our knowledge, this paper represents the first published documentation of the magnitude or distribution of anticipated errors in small-area demographic estimates by single years of age or their effects upon epidemiologic statistics. The observed magnitude of errors is large; in fact, in most cases the differences are large enough that it would be difficult to rule out average differences in risk between groups since their distributions are so likely to overlap. The range of observed errors are clearly problematic for making public health decisions. While it is obvious that scaling risk to 1,000 person years would garner substantial attention, even rescaling these statistics to 100 person years (arguably more appropriate for small-area work) does not solve this issue. For example, even if rescaled to 100 person-years a difference as large as 92/1,000 person-years would suggest a difference in risk of 9.2 persons/100 person-years. This is almost certain to trigger action by public health officials. In this respect, these results are unsettling because they suggest that errors in demographic estimates are likely to frequently have important impacts on how we utilize epidemiologic statistics for small areas. In this study, we simulated prevalence for a common condition (childhood obesity), but even after capturing a reasonable range of variation in event occurrence, the impact of demographic estimation errors was large enough to be of considerable concern.

It may be of some comfort to imagine that in the case of rarer events (such as childhood cancer, estimated to impact perhaps 1/10,000 children), the accuracy of demographic estimates should have little impact on public health decision-making. In this circumstance, even a single case of cancer should be noteworthy and a clustering of events should be identifiable indifferent of estimates of population at risk. The results presented here, however, should caution epidemiologists and public health officials of the potential uncertainties introduced by the use of demographic estimates for population at risk, though it is worth noting that using the previous decennial census counts has been shown to introduce even greater error than using postcensal estimates [8-12].

This study has assumed that epidemiologic events are captured completely. In reality, estimates of census tract-level events depend upon the process of geocoding, by which events are placed on electronic maps and then re-aggregated to summarize them at the tract level [41,53-55]. Previous studies have suggested that geocoding rates can vary from lows of 40% or less to highs approaching 90 to 95% [56-58]. These results vary across rural/urban strata and it is known that incomplete geocoding is systematic, spatially-dependent, and can bias estimates of important demographic characteristics such as race and ethnicity [42-44,58]. Haining [45] has pointed out that such incomplete geocoding is unignorable in the statistical sense [46] and a large number of studies have attempted to fill in spatially-dependent gaps in coverage through a variety of methods [6,47]. At least one study [7] has attempted to quantify the magnitude of errors introduced into small-area population estimates by incomplete geocoding. These authors suggested average errors attributable to geocoding to be approximately 9.0%, but also observed that approximately 10% of errors in total population estimates exceeded 20% and a surprising amount (nearly 4%) actually exceeded 50% error. To date, no study has estimated the impact of incomplete geocoding on estimates of age/sex structure or those with single-years-of-age, but we can expect that when postcensal estimates are used during periods between censuses rather important errors can be anticipated in both numerator (geocoding of events) and denominator (based on geocoded demographic indicators).

Temporal drift in demographic estimation accuracy should also be considered. It is largely unknown how the accuracy of demographic estimates may drift over time between censuses [8], but it is clear that it does decay over time as the time period estimating gets further away from the previous census [8]. In this study, single-year-of-age estimates were made by breaking out actual 2010 census counts in five-year age/sex-specific intervals. While it is debatable that census counts represent any sort of “gold standard” [36,39,40] it also highly plausible that they are closer to reality than any demographic or survey-based estimate can ever be at the point in time in which the enumeration takes place. In practice, demographic estimates of population at risk for five-year age/sex intervals will display their own errors, which will in turn propagate into those made for single-years-of-age. It is beyond the scope of this study to examine this drift and, in fact, any study aiming to do so is faced with the challenge that no estimates even approaching a gold standard exist for years between censuses. Ex- post-facto evaluations [8,9,41] suggest that errors in five-year age categories can be as high as 80% at the census tract level and it is unknown if these errors may offset when applied to single-year-of-age categories. For epidemiologists seeking to use demographic estimates of population at risk, postcensal drift in accuracy is a real, if immeasurable, possibility.

In spite of the potential limitations highlighted in this study, it is worth considering that alternatives may do no better and may actually be worse than using demographic estimates to capture population at risk. Previous studies have indicated that using the previous census values, for example, can produce errors that are even larger in magnitude than those observed in demographic estimates [7,8]. Not updating estimates of population at risk from the previous census is generally not advisable either and introduces an additional liability associated with not capturing changes that are important to understanding the population dynamics that ultimately produce epidemiologic risk. In terms of single-year-of-age estimates of population at risk (such as for a typical census tract of about 1,500 persons), it is likely true the number of persons within a specific age/sex interval will be small enough that even the errors observed here will have little effect on estimates of prevalence. On balance, we would argue that updating is preferred over use of the previous census. Furthermore, previous studies indicate that simple trend extrapolations (in which historical trends are carried forward) are similarly inaccurate to those produced using other methods [7-9], again recommending the use of demographic estimates for population at risk in epidemiologic statistics.

It is likely that readers of this paper will be surprised by the magnitude of error and its variability observed in this research. It is clear that errors in demographic estimates may introduce important limitations in small-area epidemiologic statistics, and this challenge has not received enough consideration in the literature. This paper should serve to spur interest in further evaluative studies as well as introducing motivation for applied demographers to resume exploration of novel methods in small-area demographic estimation in search of more accurate alternatives [7-9,59]. Both descriptive and analytic epidemiology depend upon not only accurate estimates of risk but also accounting for potential bias or uncertainty in these estimates [49,50]. From this perspective, this paper suggests that a much more detailed consideration of how error is propagated into small-area epidemiologic statistics is in order. Such an analysis must include an assessment of errors, uncertainties, and bias in both geocoding (numerator) and demographic estimates (denominator) and this paper suggests some potentially useful ways to approach this challenge.

## Competing interests

The authors have declared that they have no competing interests.

## Author’s contributions

JB, AA, and XR conceptualized the analysis. SV and NC provided data and supporting analysis. JB wrote the paper. All authors read and approved the final manuscript.
